# Cytogenetic and Molecular Data Demonstrate that the Bryconinae (Ostariophysi, Bryconidae) Species from Southeastern Brazil Form a Phylogenetic and Phylogeographic Unit

**DOI:** 10.1371/journal.pone.0137843

**Published:** 2015-09-15

**Authors:** Natália Martins Travenzoli, Priscilla Caroline Silva, Udson Santos, José Cola Zanuncio, Claudio Oliveira, Jorge Abdala Dergam

**Affiliations:** 1 Laboratório de Sistemática Molecular-Beagle, Departamento de Biologia Animal, Universidade Federal de Viçosa, CEP 36570–000, Viçosa, Minas Gerais, Brazil; 2 Instituto de Biociências, Departamento de Morfologia, Universidade Estadual Paulista (UNESP), CEP 18618–970, Botucatu, São Paulo, Brazil; Catalan Institute for Water Research (ICRA), SPAIN

## Abstract

*Brycon* spp. occur in Neotropical watersheds to the west and east of the Andes, and as they are sensitive to anthropogenic changes, many these species are endangered in southeastern Brazil. Coastal rivers in southeastern Brazil are characterized by the presence of relatively few freshwater fish species and high endemism of this fauna. The objective of this study was to examine whether *Brycon* spp. occurring in the coastal basins of southeastern Brazil are monophyletic, using cytogenetic data, mitochondrial, and nuclear molecular markers. All the species showed a diploid number of 50 chromosomes, a conserved number within the subfamily Bryconinae. However, the karyotypic formulas were unique to most species, including *Brycon devillei* (26m+22sm+2st), *Brycon ferox* (26m+12sm+12st), *Brycon insignis* (22m+20sm+8st), *Brycon opalinus*, and *Brycon vermelha* (24m+20sm+6st), indicating the prevalence of pericentric and paracentric inversions in the chromosomal evolution of these species. All of them had nucleolar organizer regions in the first pair of subtelocentric chromosomes and no equilocal distribution of heterochromatin in the first pair of chromosomes of the karyotype. These two features, not seen in any other *Brycon* spp. examined to date, indicate that Bryconinae species from the Brazilian southeastern coastal basins, including the monotypic genus *Henochilus*, are monophyletic. Also, this is the first study that reports NOR location and C-banding patterns as synapomorphies for a Neotropical fish species group. The monophyly was also supported by a phylogenetic analysis of *16S* rDNA (*16S*), cytochrome oxidase subunit I (*COI*), alpha-myosin (*MYH6*) genes and *S72* intron molecular data. Our results partially corroborate the “*Brycon acuminatus*” group proposed by Howes in 1982: our proposed clade keeps *B*. *devillei*, *B*. *ferox*, and *B*. *insignis;* but it also includes *B*. *opalinus*, *B*. *vermelha*, and *H*. *weatlandii* whereas it excludes *B*. *nattereri*. The phylogeographic unit formed by Bryconinae species in southeastern Brazil reflects the long and isolated paleohydrological history of these coastal basins relative to the continental watersheds.

## Introduction

The genus *Brycon* Müller &Troschel 1844 occurs in the watersheds that drain into the Caribbean Sea and in most of the rivers of South America [1]. The main morphological characteristics that define *Brycon* spp. are: presence of three (rarely four) series of teeth on the premaxilla, larger teeth in the inner than the outer premaxillary series, and presence of a pair of dental symphisian teeth which are uncommon in other Characidae [1]. Bryconidae are migratory fish and bioindicators of high quality habitat because they preferentially occur in rivers of clean water with high oxygen levels [1–3]. In Brazil, these species are distributed in the major river systems and are sensitive to anthropogenic changes [1, 4]. The coastal basins of southeastern Brazil are small to medium-sized watersheds characterized by the occurrence of relatively few freshwater fish species and high levels of endemism [5–7]. To the east, the coastal basins are isolated from the continental basins by the Serra do Espinhaço and Serra da Mantiqueira reliefs, the two major barriers that also represent the distribution ranges of many coastal fish populations [8, 9]. The paleohydrological history of these watersheds has been mainly influenced by local geomorphological processes and eustatic changes in sea level, which account for vicariant events and geodispersal processes affecting the freashwater fish faunas [8–10].

To date, no phylogenetic studies have encompassed all Bryconinae from the coastal basins of southeastern Brazil; previous studies have included *Brycon ferox*, *Brycon opalinus*, *Brycon insignis*, *Brycon vermelha* and *Henochilus wheatlandii* [11–14]. Molecular phylogenies based on mitochondrial and nuclear DNA sequences have already revealed the paraphyletic condition of *Brycon*, as some coastal species of this genus are phylogenetically closely related to *H*. *wheatlandii* [11, 14].

A morphological group comprising bryconins from southeastern Brazil was suggested by Howes [2], who proposed that *Brycon* might be divided into five groups, two are trans-Andean and three of them are cis-Andean. The trans-Andean groups are the group *Brycon alburnus* and *Brycon guatemalensis*. The former is composed of *Brycon alburnus* and *Brycon atrocaudatus* and the latter is composed of *B*. *guatemalensis*, *Brycon meeki*, *Brycon oligolepis*, *Brycon striatulus*, and *Brycon rubricauda*. The cis-Andean groups are *Brycon falcatus*, *Brycon orbignyanus* and *Brycon acuminatus*. The first group is represented by *Brycon amazonicus*, *Brycon bicolor*, *Brycon cephalus*, *B*. *falcatus*, *Brycon hilarii*, *Brycon moorei*, *Brycon orthotaenia*, and *Brycon bahiensis* (later synonymized to *B*. *opalinus*);. The second group is composed of *B*. *hilarii* and *B*.*orbignyanus;* finally, the third group is composed of *B*. *insignis* (Howes’ *B*. *acuminatus*), *B*. *ferox*, *Brycon reinhardti* (later synonymized to *B*. *nattereri*), and *B*. *devillei*. The latter group included many southeastern Brazilian bryconins. Besides *B*. *nattereri*, that occurs in the Upper Paraná Basin, the *B*. *acuminatus* group species share some morphological characters such as long maxilla with many teeth, small dental, a simple color pattern characterized by humeral and caudal spot, and long and pointed snout. With the exception of the humeral spot, *B*. *vermelha* can be included in this group because it has all the other characters [4]. Although Howes [2] did not indicate that "*Brycon acuminatus*" is monophyletic, Lima and Castro [4] suggested that *B*. *vermelha* is a member of that group, and that the *Brycon* that occur in the southeastern Brazil might be a monophyletic group based on morphological characters.

Few studies on Neotropical freshwater fish species have been conducted to test phylogeographic hypotheses with cytogenetic marks [10, 13, 15, 16]. Most cytogenetic studies on Neotropical fishes aimed detecting cryptic species and chromosomal evolution in large taxonomic groups. These studies have routinely applied two classic chromosome banding techniques (Ag-NOR and C-banding) because they are cost-effective for characterization of numerical and structural chromosomal alterations. The Ag-NORs technique allows to identify nucleolus organizer regions (NORs) active on the last cell interphase and involves silver nitrate precipitation on proteins involved in the transcription of rDNA cistrons with 18S rDNA [17]. The C-banding technique allows to identify constitutive heterochromatin regions and it involves differential degradation of euchromatic and heterochromatic chromosomal regions by alternating treatments with acids and bases [18]. Together, these techniques have successfully contributed to our knowledge of the chromosomal evolution of Neotropical freshwater fishes [16, 19, 20 21, 22, 23, 24].

Cytogenetic studies on Brazilian coastal species of *Brycon* have been restricted to Paraíba do Sul River basin populations of *Brycon insignis* [22]. In continental watersheds, cytogenetic studies have been carried out in *Brycon* spp. from the Amazon River Basin (*B*. *cephalus*), Paraguay River Basin (*Brycon hilarii*), Tocantins River Basin (*B*. *falcatus*), Magdalena-Cauca River Basin (*Brycon henni*), Paraná River Basin (*B*. *orbignyanus*), Orinoco River Basin (*Brycon amazonicus*) and São Francisco River Basin (*B*. *orthotaenia*) [22–26]. A study on “*B*. *nattereri*” from the Paraíba do Sul Basin [22] seems to be a misplacement or misidentification, because this species is restricted to the Upper Paraná Basin. All studies have indicated a conserved diploid number of 50 chromosomes and a karyotype composed of metacentric chromosomes (characterized by the median location of the centromere and chromosome arms of similar length); submetacentric chromosomes (characterized by the displaced position of the centromere and rather unequal chromosome arms), and subtelocentric chromosomes (characterized by a centromere that is much closer to one of the chromosome telomeric regions and very unequal chromosome arms) [27]. Patterns of chromosome evolution that do not alter the diploid number are usually interpreted as the outcome of chromosome rearrangements called pericentric inversions (chromosome break/fusions that occur around the chromosome’s centromere) and paracentric inversions (chromosome break/fusions that occur within the same chromosome arm) that do not involve the centromere. Because they alter the chromosome morphology, pericentric inversions alter the chromosome formulae, whereas paracentric inversions keep the chromosome morphology [19, 28, 29]. Also, all these *Brycon* spp. have their NORs in the terminal region of the long arm of the second pair of submetacentric chromosomes [22, 23, 25–28, 30]. Based on the distribution patterns of heterochromatin, Margarido and Galetti Jr. [23] proposed the existence of two groups of *Brycon*. The first group is characterized by heterochromatic pericentromeric markings, predominantly on the submetacentric chromosomes, whereas the second group shows heterochromatic blocks on the telomeres of metacentric chromosomes, and the presence of a distinct equilocal heterochromatic block on the first pair of metacentric chromosomes. A third, divergent pattern occurs in the coastal bryconin *H*. *wheatlandii*, which has a pair of NORs located in a pair of subtelocentric chromosomes, heterochromatic blocks in subtelocentric chromosomes, and non-equilocal heterochromatin in its first chromosome pair [13]. Thus, based on the cytogenetic differences between *H*. *wheatlandii* and other Bryconinae, we hypothesized that at least some of these characteristics could also be shared with other Bryconinae from southeastern Brazil, suggesting the existence of a phylogeographic unit within this subfamily. The aim of this study was to examine this hypothesis with a combined approach using cytogenetic data, fragments of mitochondrial DNA (cytochrome oxidase subunit I–*COI* and *16S* rDNA) and two nuclear DNA fragments (the cardiac muscle myosin heavy chain 6 alpha-*MYH6* gene and the *S72* intron) on six southeastern bryconins: *B*. *devillei*, *B*. *ferox*, *B*. *insignis*, *B*. *opalinus*, *B*. *vermelha*, and *H*. *wheatlandii*.

## Materials and Methods

### Sampling, Preparation of Chromosomes, Banding, and Karyotypic Analysis

**Fig 1:**
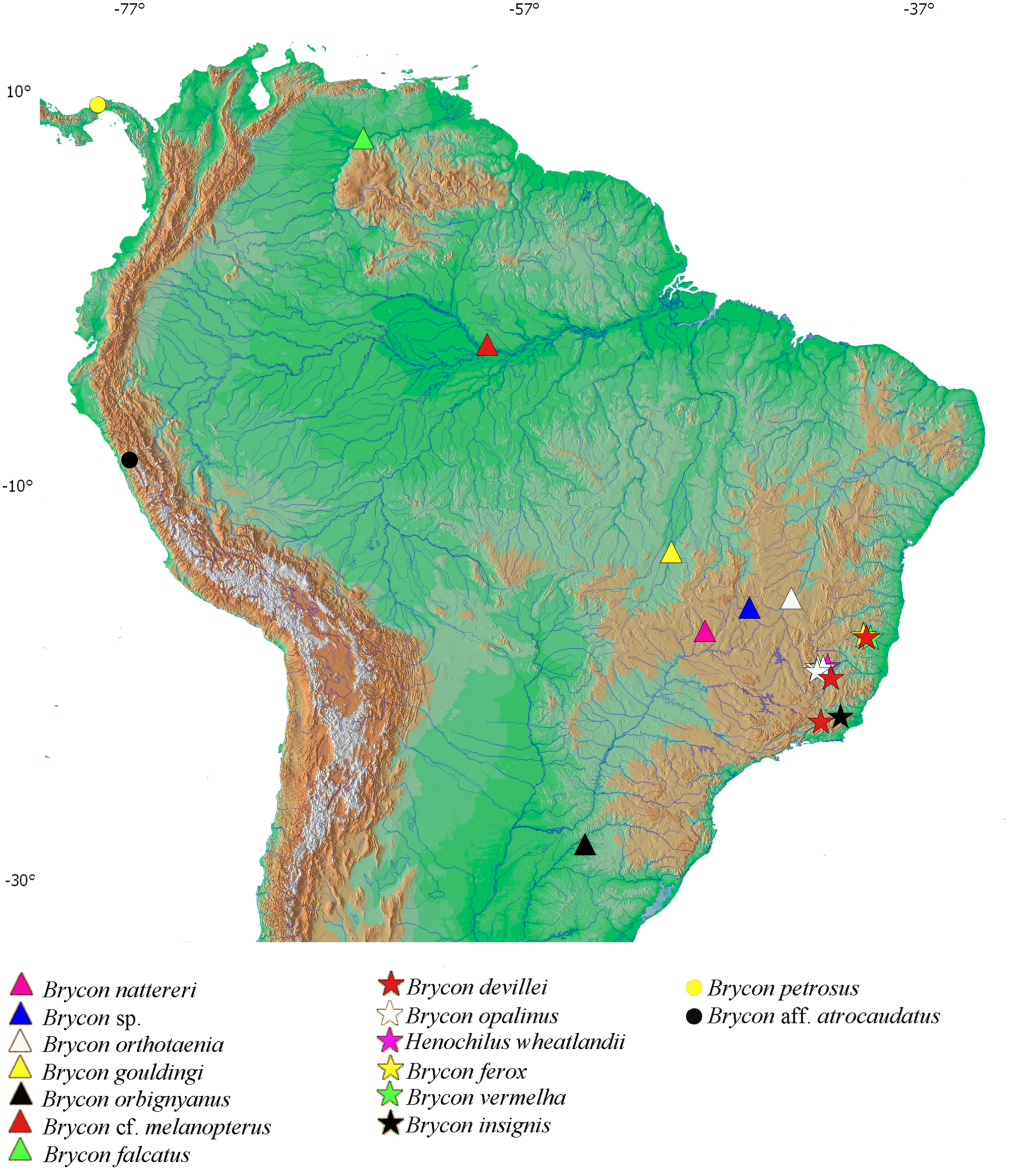
Coastal and continental basins in southeastern Brazil and collect local of the Bryconine species examined on this study.

A total of 78 samples of *Brycon* spp. were collected on three coastal basins of eastern Brazil (Fig 1) and were compared with other species from seven South American river basins comprising four of Howe’s bryconine groups (Table 1). The collections were carried out with collecting permit from the Instituto Chico Mendes de Biodiversidade (ICMBio) (SISBIO14975-1) issued to JAD. The specimens were deposited in the João Moojen de Oliveira Museum of Zoology at the Universidade Federal de Viçosa, Viçosa, Minas Gerais State, Brazil (MZUFV3564, MZUFV3969, MZUFV4008, MZUFV4012, MZUFV4027, MZUFV4049-4051, MZUFV4092 and MZUFV4144), Universidade Federal do Rio Grande do Sul, Porto Alegre, Rio Grande do Sul State, Brazil (UFRGS12687, UFRGS17127 and UFRGS11377) and Universidade Estadual Paulista, Botucatu, São Paulo State, Brazil (LBP13782; LBP4211; LBP2750; LBP12818; LBP3130; LBP3027; LBP1356).

**Table 1:** Bryconin species, number of samples used on cytogenetic and molecular analyses with geographical coordinates of sampling sites.

**Species**	**Sample size**			**GPS coordinates**	**Locality (Hydrographical basin)**
	**Cytogenetics**		**Molecular**		
	♂ **♀**				
*Brycon amazonicus*	00	00	02	21° 58'07"S 43° 07'43"W	Doce River, Santana do Deserto, MG (Doce River basin—Brazil).
*Brycon aff*. *atrocaudatus*	00	00	01	08°40'40''S78°09'163''W	Rio Santa (Pacific—Peru).
*Brycon devillei*	01	00	01	19°45'24"S 42°37’13"W	Carioca Lake, Dionísio, MG (Doce River basin—Brazil).
	06	05	03	21° 58'07"S 43° 07'43"W	Doce River, Santana do Deserto, MG (Doce River basin—Brazil).
	00	02	02	17°41'09"S 40°50'33"W	Mucuri River, Carlos Chagas, MG (Brazil).
*Brycon falcatus*	00	00	01	07°38'11.6''S 66°19'04.2'' W	Orinoco River, Caicara del Orinoco (Venezuela)
*Brycon ferox*	04	03	02	17°41'09"S 40°50'33"W	Mucuri River, Carlos Chagas, MG (Mucuri basin—Brazil).
*Brycon gouldingi*	00	00	01	13°20'051''S50°42'162'W	Lagoa da Égua, Mato Grosso (Araguaia River basin—Brazil)
*Brycon insignis*	07	02	02	21°42'35"S 42°07'55"W	Paraíba do Sul River, Itaocara, RJ (Paraíba do Sul River basin—Brazil).
*Brycon* cf. *melanopterus*	00	00	01	28°37'44"S 60°58'44"W	Balneary Adão e Maria, Manaus, AM (Amazonas River basin—Brazil).
*Brycon moorei*	00	00	01	not availabe	Magdalena River, Antioquia, (Colombia).
*Brycon nattereri*	00	00	02	17°20'00"S 49°0'05"W	Córrego Coqueiro, Cachoeira das Piracanjubas, GO (Paraíba do Sul/Alto Paraná Rivers basin—Brazil).
*Brycon opalinus*	01	02	02	19°13'02"S 42°53'03"W	Santo Antônio River, Sete Cachoeiras, MG (Doce River basin—Brazil).
	07	03	00	19°13'24"S 42° 52'12"W	Esmeralda Stream, Sete Cachoeiras, MG (Doce River basin—Brazil).
	02	02	02	19°25'11"S43°19'22"O	Preto/Itambé do Mato Dentro River, MG (Doce River basin—Brazil).
**Species**	**Sample size**			**GPS coordinates**	**Locality(Hydrographicalbasin)**
	**Cytogenetics**		**Molecular**		
	♂**♀**				
*Brycon orthotaenia*	00	00	02	15°40'18"S 44° 37"43"O	River Pandeiros, Januária, MG (São Francisco River basin—Brazil).
*Brycon orbignyanus*	00	00	01	28°08'33''S 55°04'44''O	River Ijuí, Roque Gonzales, RS (Uruguai River—Brazil).
*Brycon petrosus*	00	00	01	09°19'262''N79°46'082''O	Río Llano Sucio, Panama (Atlantic)
*Brycon* sp.	00	00	01	16°26'66"S 46°12'67"O	Palmeirinha, MG (São Francisco River basin—Brazil).
*Brycon vermelha*	04	02	02	17°41'09"S 40°50'33"O	Mucuri River, Carlos Chagas, MG (Mucuri River basin—Brazil).
*Henochilus wheatlandii*	00	00	01	19°13'02"S 42°53'03"O	Santo Antônio River, Sete Cachoeiras, MG (Doce River basin—Brazil).

The collected specimens were anesthetized with clove oil at a concentration of 0.3 gL^−1^ [31], as approved by the Universidade Federal de Viçosa Ethics Committee (permit 032/2013). Mitotic chromosomes were obtained from the anterior kidney of fishes collected on coastal river basins, following Bertollo *et al*. [32], stained by conventional staining (Giemsa), and classified according to their arm ratio (longer chromosome arm/shorter chromosome arm) in metacentrics (1,00–1,69), submetacentrics (1.70–2.99) and subtelocentrics (3,00–6,99) following Levan *et al*. [33].

The NORs that were active in the last cell interphase were identified using silver nitrate precipitation [17]. The morphology of the NOR-bearing chromosome (S1 Fig) was estimated from using the arms ratio of 10 metaphase plates per specimen. The regions of constitutive heterochromatin were evidenced using C-banding [18]. The images of metaphases were obtained with a Olympus BX53 microscope with Olympus CellSens Imaging Software and measured using Image Pro Plus^®^ software.

### DNA Extraction, Amplification, and Sequencing

The DNA was extracted from the gill filaments, muscle, or liver tissue of the samples, and fixed in 95% ethanol following Boyce *et al*. [34]. The *COI* gene was amplified with primers cocktail FishF1t1 and FishR1t1 [35, 36], the *16S* gene was amplified with primers Sar-5and Sbr-3 [37], the *MYH6* gene was amplified with nested-PCR using the primers F459 and R1325 (1^st^ PCR) and F507 and R1322 (2^nd^ PCR) [38] and the *S72* gene was amplified with primers S72F and S73R [39]. The PCR reactions for COI and 16S genes were carried out in a reaction volume of 12.5 μL [8.76 μL of H_2_0, 1.2 μL of 10× reaction buffer (200 mM Tris-HCl and 500 mM KCl; pH 8.4), 0.3 μL of MgCl_2_ (100 mM), 0.05 μL of dNTPs (20 mM), 0.12 μL of each primer (10μM), 0.0625 μL (2.5 U) of *Taq* polymerase (Phoneutria®), and 200 ng of template DNA]. For *COI* and *16S* reaction (PCR) conditions were as follows: 94°C (2 min), and 35 cycles of 94°C (30 s), 52°C (40 s), and 72°C (1 min), and 72°C (10 min). PCR reactions for *MYH6* and *S72* intron were carried out in a reaction volume of 20 μL [10.3 μL of H_2_ 0, 2 μL of 10× reaction buffer (Platinum® *Taq*—Invitrogen), 0.6 μL of MgCl_2_ (50 mM), 2 μL of dNTPs (2 mM), 2 μL of each primer (2 μM), 0.1 μL (5 U) of Platinum® *Taq*, and 100 ng of template DNA]. For *MYH6*, the first PCR was as follows: 94°C (3 min), 35 cycles of 94°C (30 s), 53°C (45 s), 72°C (1 min and 30 s), and 72°C (10 min). The second PCR was: 94°C (3 min), 35 cycles of 94°C (30 s), 62°C (45 s), 72°C (1 min and 30 s), and 72°C (5 min). For *S72*, PCR conditions were as follows: 95°C (2 min), 35 cycles of 95°C (30 s), 54°C (30 s), 72°C (1 min), and 72°C (10 min). The PCR products were purified by using enzimatic method Exosap (25% exonuclease, 25% Shrimp Alkaline Phosphatase and 50% of deionized water) or PEG 8000 (20% polyethyleneglycol, 2.5 M NaCl), and sequencing was performed on a Macrogen sequencing platform of Macrogen, Seoul, South Korea. Sequences of each locus were independently aligned using Clustal W [40] with sequencing chromatograms checked by eye and alignments realigned with Muscle [41] in MEGA 5.0 software [42]. Standard genetic summaries were calculated for each gene using MEGA 5.0 [42].

DNA alignments were concatened and analysed using a partitioned Bayesian approach. The molecular evolution models were selected based on the Bayesian information criterion BIC in Partition Finder v1.1.0 [43]. The data set was divided into eight sections corresponding to 16S gene and S72 intron and first, second and third positions for the genes COI and MYH6. Bayesian inference was performed two times by using MrBayes software version 3.2.3 [44] with four independent Markov chain Monte Carlo (MCMC) that were run 30,000,000 replicates with a phylogenetic tree sampled every 1,000 generations. The first 25% generations were discarded as burn-in and the remaining trees were used to generate statistics and topology. The distribution of log likelihood scores was examined to determine stationarity for each search and to decide whether extra runs were required to achieve convergence using the program Tracer 1.4 [45].

Maximum likelihood analysis was performed with RAxML version 8.1.1[46], using a mixed partition model indicated by Partition Finder v1.1.0 [43] with all parameters set to default values. Topological robustness was assessed using 1,000 nonparametric bootstrap replicates. MrBayes and RAxML analyses were run on computational resources provided by Cyberinfrastructure for Phylogenetic Research (CIPRES) [47].

Maximum parsimony analyses were conducted with the concatenated alignment within PAUP* 4.0b10 [48]. Heuristic searches were performed with 100 random addition replicates and TBR branch swapping. All characters were unordered, all character transformations were equally weighted, and gaps were treated as missing data. Clade robustness was verified with 1,000 bootstrap pseudoreplicates [49]. The posterior probability values of 1–0.91 output by MrBayes and bootstrap values over 90% output by RAxML and maximum parsimony analyses were considered as well supported [50]. The sequences amplified in this study were deposited in GenBank (Access No. XXXX).

## Results

### Karyotypic Analyses

**Fig 2:**
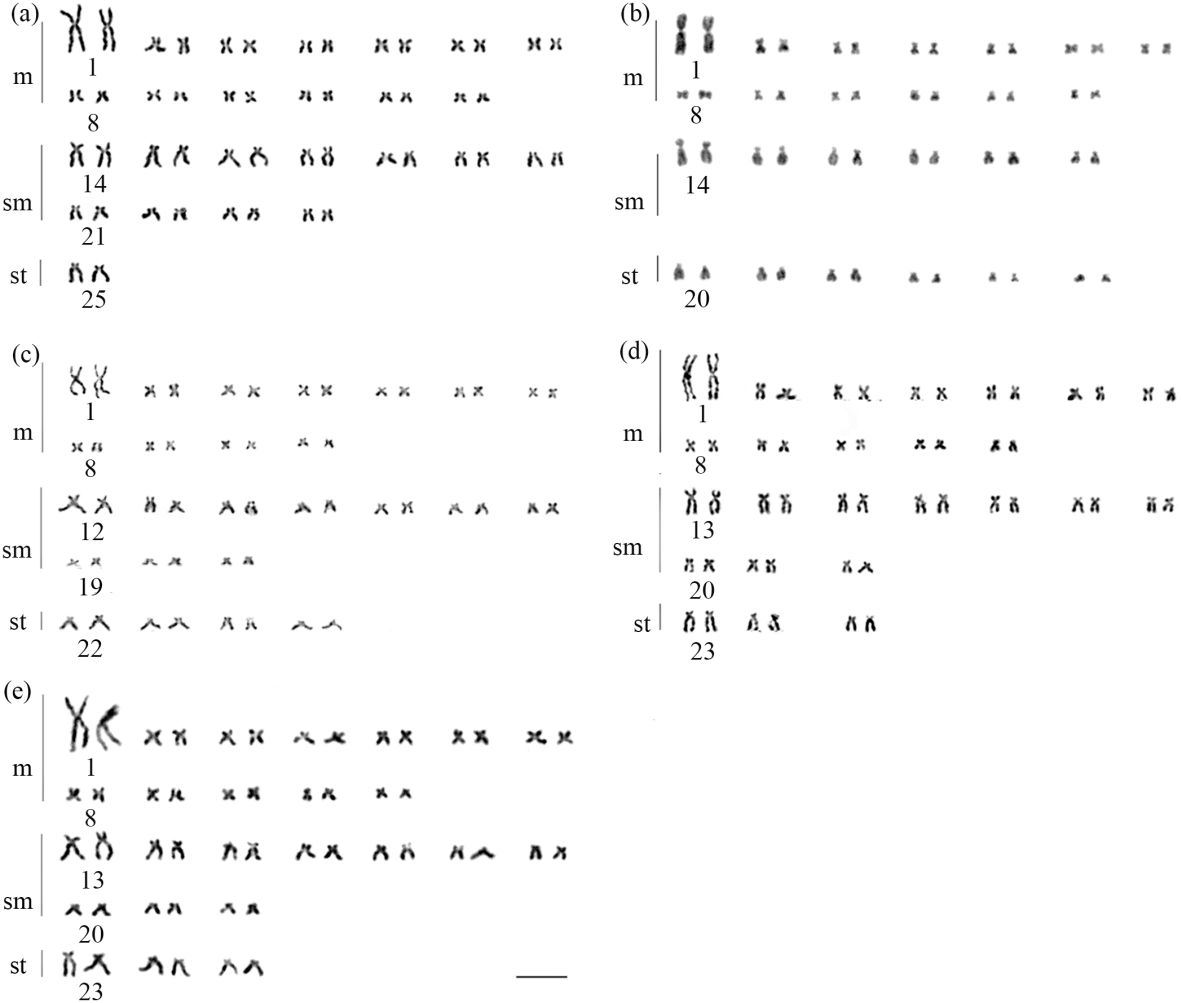
Karyotype of *Brycon* species from eastern Brazil. *Brycon devillei* (a), *Brycon ferox* (b), *Brycon insignis* (c), *Brycon opalinus* (d), and *Brycon vermelha* (e). The bar represents 10 μm.

**Fig 3:**
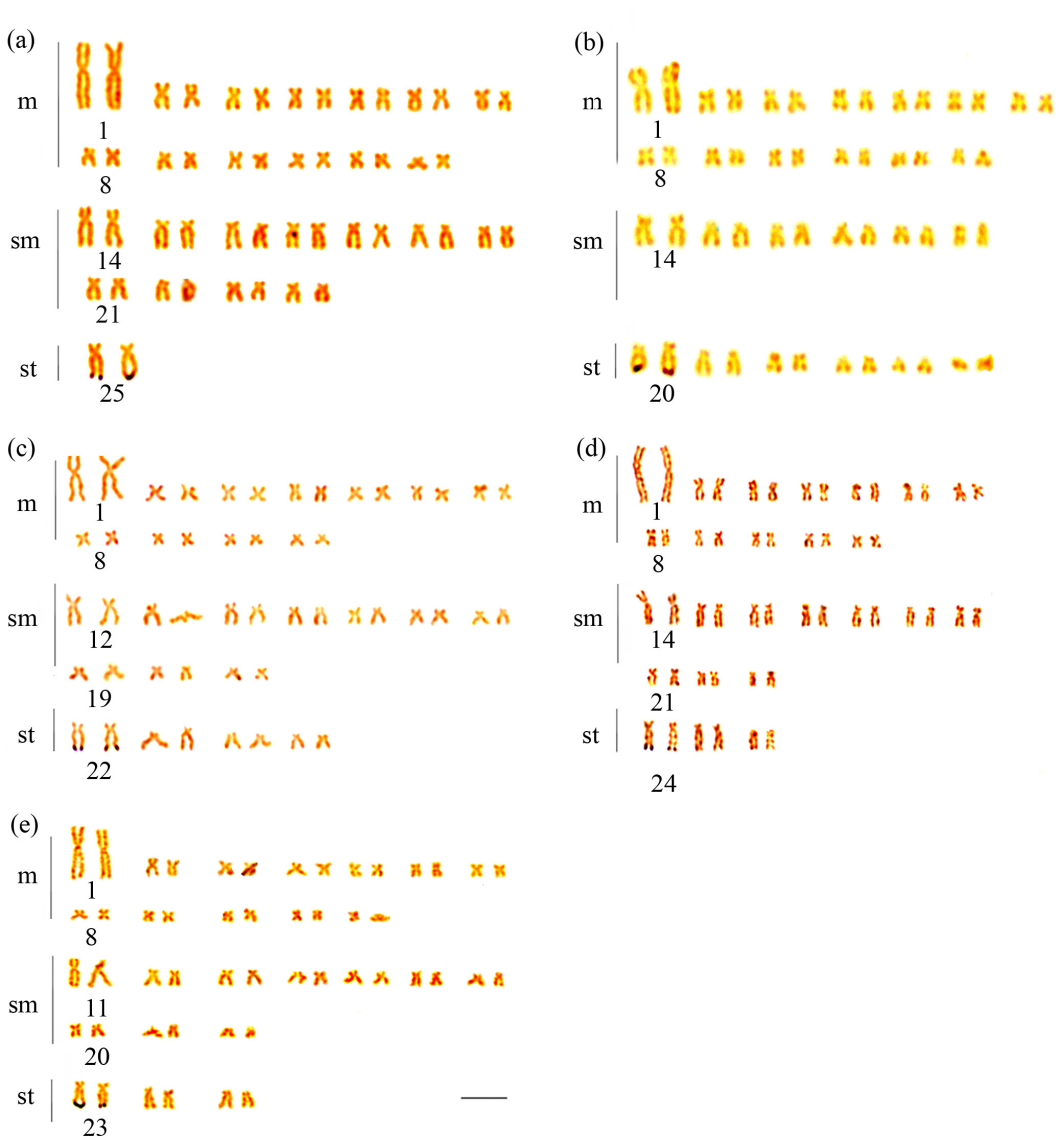
Distribution of active NORs in the last cell interphase in *Brycon* speciesthat occur in the basins of eastern Brazil. *Brycon devillei* (a), *B*. *insignis* (b), *B*. *ferox* (c), *B*. *opalinus* (d), and *B*. *vermelha* (e). The bar represents 10 μm.

**Fig 4:**
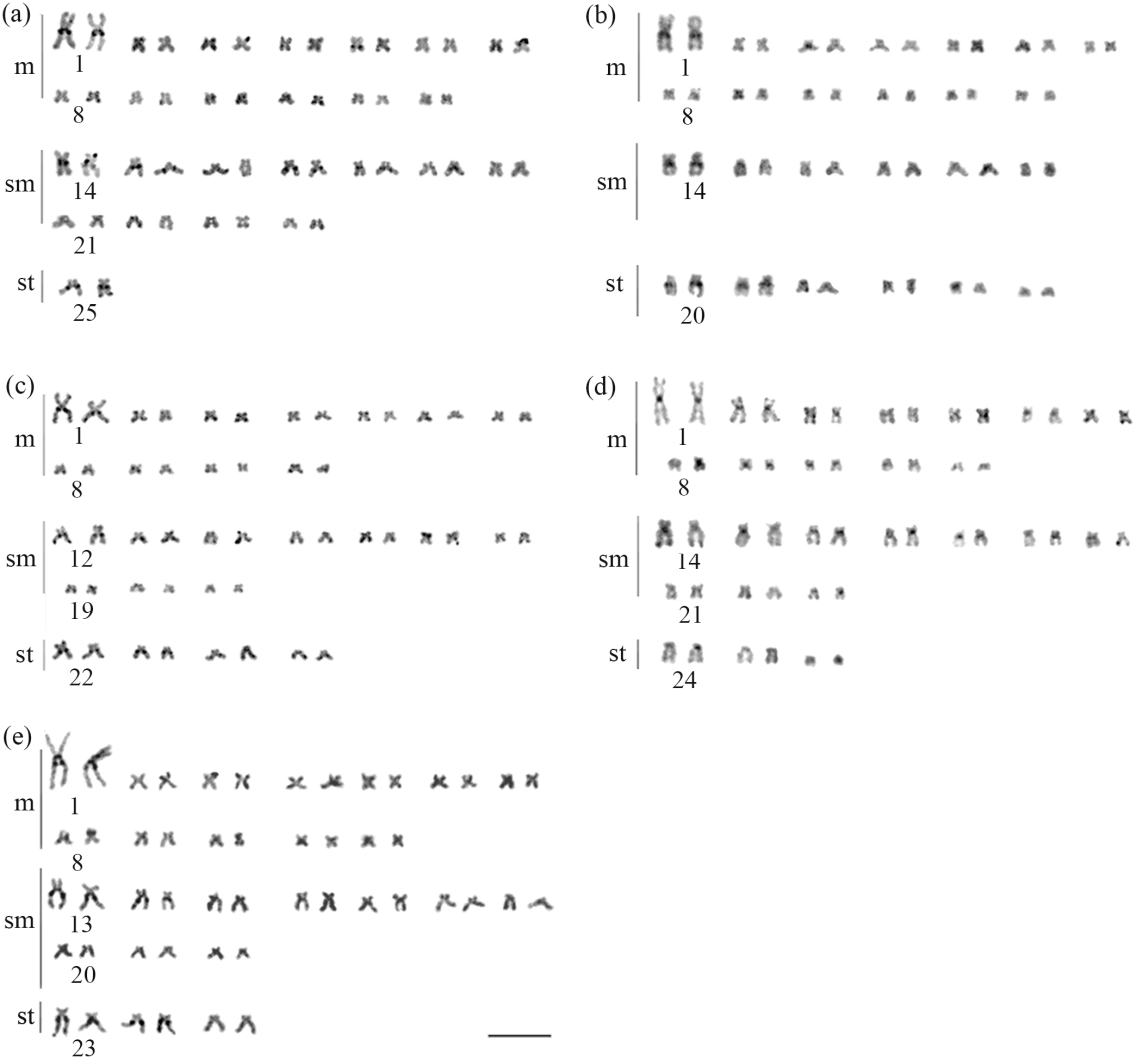
Heterochromatic patterns of *Brycon* species that occur in the basins of eastern Brazil. *Brycon devillei* (a), *B*. *insignis* (b), *B*. *opalinus* (c), *B*. *ferox* (d), and *B*. *vermelha* (e). The bar represents 10 μm.

All species of *Brycon* from the coastal basins fo southeastern Brazil had diploid number of 50 chromosomes and a fundamental number equal to 100, though the karyotypic formulae varied among most of the species; no polymorphisms were observed within each species: *B*. *devillei*, 26m+22sm+2st; *B*. *ferox*, 26m+12sm+12st; *B*. *insignis*, 22m+20sm+8st; and *B*. *opalinus* and *B*. *vermelha*, 24m+20sm+6st (Fig 2). In all samples, NORs occurred in the terminal region of the long arm on the first pair of subtelocentric chromosomes (Fig 3). All samples exhibited a pericentromeric heterochromatic block on the long arm of the first pair of the metacentric chromosomes. Additionally, in *B*. *vermelha* a centromeric heterochromatin block was also observed on the first chromosome pair (Fig 4). On the other hand, the presence of pericentromeric heterochromatic regions varied among samples and characterized different chromosome pairs: *B*. *devillei*: 14, 15, 17, 18, and 25 (Fig 4a); *B*. *ferox*: 14, 20, and 21 (Fig 4b); *B*. *insignis*: 12,13, 22, 23, and 24 (Fig 4c); *B*. *opalinus*: 14, 15, 16, 18, and 20 (Fig 4d); and *B*. *vermelha*: 13, 14, 23, and 24 (Fig 4e) (Table 2).

**Table 2:** Bryconin species, karyotypic formulae, Ag-NORs and heterochromatic patterns.

**Species**	**Karyotypic formulae**	**Ag-NORs**	**Heterochromatic patterns**
*Brycon devillei*	26m+22sm+2st	On terminal region of the long arm of the first pair of subtelocentric chromosomes.	Pericentromeric block on the long arm of the first pair of the metacentric chromosomes.
*Brycon ferox*	26m+12sm+12st	On terminal region of the long arm of the first pair of subtelocentric chromosomes.	Pericentromeric block on the long arm of the first pair of the metacentric chromosomes.
**Species**	**Karyotypic formulae**	**Ag-NORs**	**Heterochromatic patterns**
*Brycon insignis*	22m+20sm+2st	On terminal region of the long arm of the first pair of subtelocentric chromosomes.	Pericentromeric block on the long arm of the first pair of the metacentric chromosomes.
*Brycon opalinus*	24m+20sm+6st	On terminal region of the long arm of the first pair of subtelocentric chromosomes.	Pericentromeric block on the long arm of the first pair of the metacentric chromosomes.
*Brycon vermelha*	24m+20sm+6st	On terminal region of the long arm of the first pair of subtelocentric chromosomes.	Pericentromeric block on the long arm of the first pair of the metacentric chromosomes and centromeric block was also observed on the first chromosome pair.

### Molecular Analyses

Sequence alignment of the 418-bp *16S* gene fragment yielded 100 variable sites, 62 of them were parsimony informative; the average nucleotide composition was *π*_T_ = 0.20; *π*_C_ = 0.24, *π*_A_ = 0.34, and *π*_G_ = 0.22. The estimated average transition/transversion rate was 4. Sequence alignment of the 599 bp- *COI* gene fragment was obtained for all species, except for *Brycon melanopterus*, which was included as missing data in the multiple alignments. This fragment yielded 193 variable sites, 147 of them were parsimony informative, the average nucleotide composition was *π*_T_ = 0.30, *π*_C_ = 0.27, *π*_A_ = 0.25, and *π*_G_ = 0.18. The estimated average transition/transversion rate was 9. The 733 bp-long fragment of the *MYH6* gene was obtained for most samples, except *Brycon atrocaudatus* and *Brycon petrosus*; those sequences were considered missing data in the multiple alignments. The *MYH6* fragment yielded 53 variable sites, 30 of them were parsimony informative, and the average nucleotide composition was *π*_T_ = 0.24, *π*_C_ = 0.22, *π*_A_ = 0.30, and *π*_G_ = 0.24. The estimated average transition/transversion rate was 2.

The *S72* 356 bp-long fragment was obtained for most samples, except for *Brycon atrocaudatus*, which was included as missing data in the multiple alignment. The S72 fragment yielded 172 variable sites, 77 of them were parsimony informative, and the average nucleotide composition was *π*_T_ = 0.34, *π*_C_ = 0.19, *π*_A_ = 0.26, and *π*_G_ = 0.21. The estimated average transition/transversion rate was 11. Twelve heterozygote sites were observed among the 32 specimens and the nucleotide code followed the International Union of Pure and Applied Chemistry (IUPAC). The final matrix with DNA sequences concatenated resulted in 2,109 bp fragment deposited in TreeBase (www.treebase.org) under number XXXX.

The most suitable partitions indicated by Partition Finder was composed of five subsets: (1) molecular model K80+I+G for *16S* gene partition, *COI*_1^st^ codon position and *MYH6*_3^rd^ codon position; (2) molecular model F81 for *COI*_2^nd^ codon position; (3) molecular model GTR+G for *COI*_3^rd^ codon position; (4) molecular model F81+I for *MYH6*_1^st^ and *MYH6*_2^nd^ codon position, and (5) HKY+G for *S72* partition.

**Fig 5:**
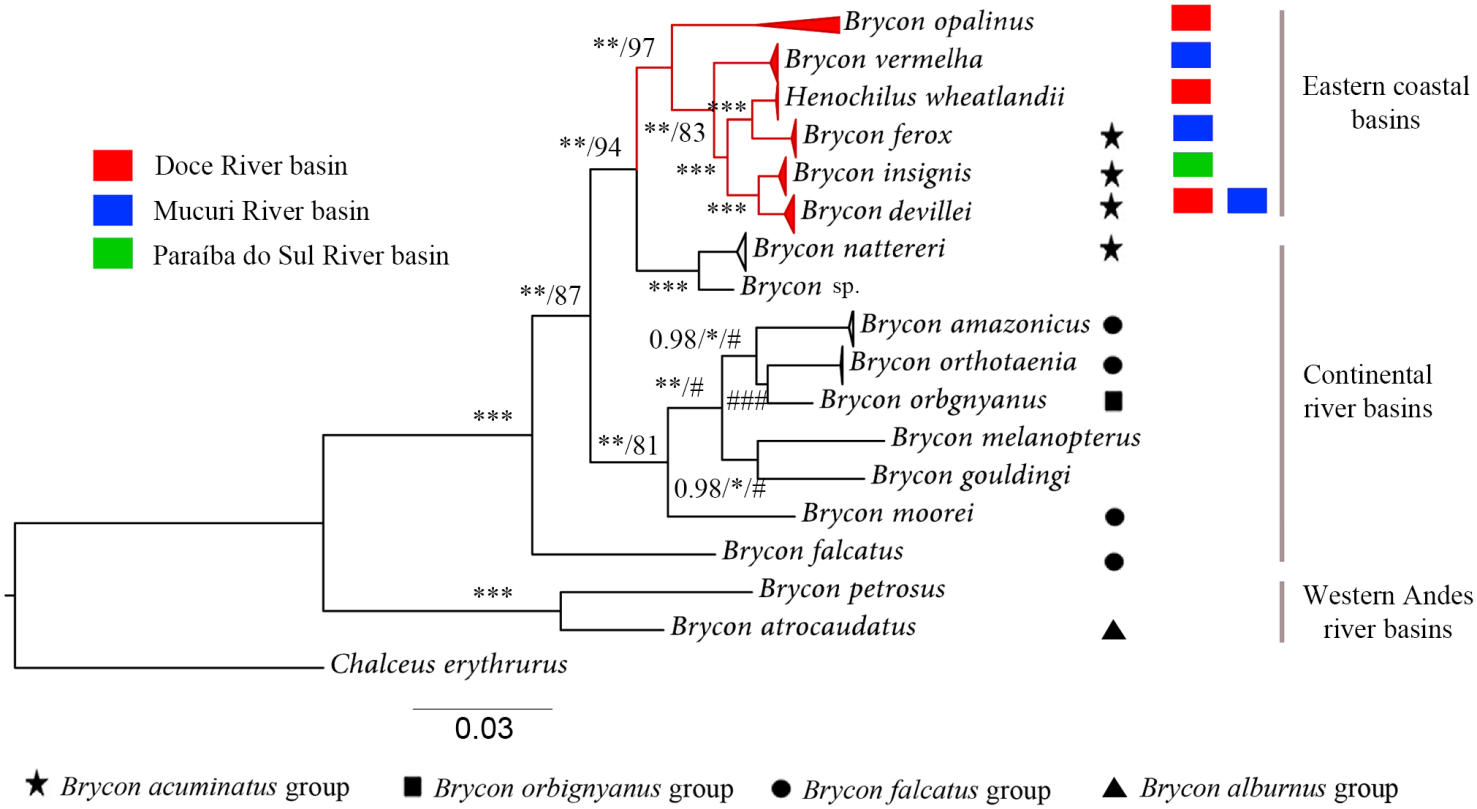
Hypothesis of phylogenetic relationships among some species of the subfamily Bryconinae. The numbers and symbols at nodes indicate the posterior probability (first) and bootstraps (second and third) obtained by Bayesian, maximum likelihood and maximum parsimony approaches, respectively. * denotes posterior probability = 1 and bootstrap values = 100. # denotes posterior probability lower than 0.9 and boostrap lower than 80%. Symbols indicate three cis-Andean and one trans-Andean morphological groups proposed by Howes (1982). The “*B*. *guatemalensis*” species group were not included in the analyses. Painted squares denote the coastal basins of eastern Brazil where samples were collected. Bars represent the molecular distance.

The phylogenetic hypothesis obtained with concatenated genes with Bayesian inference (MrBayes), maximum likelihood (RAxML) and maximum parsimony (PAUP) recovered two groups to the east and to the west of the Andean relief. Within the eastern Andean group, one sub-group was formed by the coastal species *B*.*insignis*, *B*. *devillei*, *B*. *ferox*, *H*. *wheatlandii*, *B*. *vermelha*, and *B*. *opalinus* (Fig 5).

## Discussion

Karyotypes composed of 2*n* = 50 metacentric, submetacentric, and subtelocentric chromosomes characterize the Bryconinae [13, 22, 23, 25–27, 51] and *Salminus* species [27, 52, 53]. The close phylogenetic relationship between *Brycon* spp. and *Salminus* spp. led some researchers to propose the Family Bryconidae [12]. Despite the stable diploid number, the differences in the chromosomal formulae among *Brycon* spp. from southeastern Brazil indicate a preponderance of pericentric and possible paracentric inversions in the karyotypic evolution of this group. The stable karyotypical macrostructure and the basal position of *B*. *opalinus* and *B*. *vermelha* suggest that their karyotypic formula of 24m+20sm+6st is a plesiomorphy in southeastern Brazilian bryconins. Pericentric inversions occurred in the ancestor of *H*. *wheatlandii* and *B*. *ferox* resulting in the chromosome formula of 26m+12sm+12st shared by these two species. Finally, the dichotomy between *B*. *devillei* and *B*. *insignis* involved the retention of 26 metacentrics in *B*. *devillei* and alteration in the number of submetacentrics and subtelocentrics, whereas the karyotype of *B*. *insignis* involved changes in the number of metacentrics, submetacentrics, and subtelocentrics. Pericentric inversions are relevant in the speciation of several groups of animals, including fish [19], insects [54, 55], and reptiles [56], and may represent potential isolation factors among among sympatric fishes with similar reproductive habits.

The presence of NORs in the terminal regions of the long arms of the first subtelocentric pair of chromosomes in the southeastern *Brycon* spp. is similar to that previously reported in *H*. *wheatlandii* [13], corroborating a second pattern that diverged from other Neotropical Bryconinae such as *B*. *cephalus*, *B*. *orthotaenia*, *B*. *hilarii*, and *B*. *orbignyanus*, which have a pair of NORs on the second submetacentric chromosome pair [22, 23, 26]. Although Almeida-Toledo *et al*. [22] and Margarido and Galetti Jr. [23] indicate that *B*. *insignis* is characterized by NORs in a pair of submetacentric chromosomes, the *B*. *insignis* sample included in the present study showed sites of NORs in its first pair of subtelocentric chromosomes. This difference suggests the existence of polymorphism in this species, because both samples were collected in the Paraíba do Sul River Basin. One possible exception for the presence of NORs in the subtelocentric chromosomes in the continental species is *B*. *amazonicus* [25]. Although Mariguela *et al*. [25] indicate that this species has its NORs in a subtelocentric chromosome pair, these authors included the NOR-bearing chromosome in the submetacentric chromosome group. Therefore, the presence of NORs in a subtelocentric chromosome pair is restricted to Bryconinae spp. from the coastal basins of southeastern Brazil.

Another unique feature of the eastern coastal bryconine is the distribution pattern of heterochromatic blocks in the chromosomes. All *Brycon* species present in the southeastern Brazilian coastal watersheds, including *H*. *wheatlandii*, lack equilocal heterochromatic blocks in their first chromosome pair, whereas *Brycon* species occurring the continental watersheds have heterochromatic equilocal blocks [23, 25, 27]. Indeed, the presence of equilocal heterochromatic blocks in *B*. *amazonicus*, *B*. *hilarii*, *B*. *orthotaenia*, and *Salminus hilarii* may indicate a plesiomorphic feature within the Bryconidae [12, 25, 27]. Consequently, the loss of equilocality is a synapomorphy that characterizes the southeastern coastal bryconins, whereas the presence of heterochromatic block in the telomeric region of the first pair is an autapomorphy in *H*. *wheatlandii* [13].

As previously indicated, the variation in heterochromatine patterns among species of *Brycon* were used by Margarido and Galetti Jr. [23] to propose the existence of two species groups within this taxon. The first group includes the species *B*.*cephalus*, *B*. *hilarii*, and *B*. *orbignyanus*" *that revealed telomeric bands in* ***some*** *metacentric chromosomes*, " whereas the second group was characterized by having "***predominantly*** *centromeric and pericentromeric positive C band*, ***mainly*** *in submetacentric chromosomes*". This second group is represented by *B*. *orthotaenia*, *B*. *falcatus*, and *B*. *insignis*. A putative third pattern of heterochromatin was proposed for *H*. *wheatlandii*, which was characterized by telomeric marks on the first metacentric chromosome pair and predominantly pericentromeric markings in the subtelocentric chromosomes [13]. Silva *et al*. [13] hypothesized that bryconine occurring in the coastal basins of eastern Brazil might share the third pattern, which is supported by the results obtained in the present study.

To date, the cytogenetic data of Neotropical fishes have shown some major trends: karyotypic stability in Anostomidae [57, 58], Prochilodontidae [58, 59–61], and Curimatidae [57, 62], and the existence of species complexes in the genera *Astyanax* [63] and *Hoplias* [64]. However, the cytogenetic pattern observed in bryconine from Brazilian southeastern coastal basins represents the first case in which synapomorphies of chromosomal banding show monophyly of well-defined morphological species.

A well-supported clade formed by bryconine from eastern Brazilian coastal river basins was recovered in the molecular analyses. This topology differs from the one proposed by Abe *et al*. [14] were *Brycon nattereri* appears as more related to the eastern coastal basins brycone than *Brycon opalinus*. Species sampling, such as the inclusion of *B*. *devillei* and the use of genes with faster substitution rates may have influenced this result. Long branches and low boostrap values using maximum likelihood and maximum parsimony analyses were shown on the ancestral node shared by *B*. *nattereri* and other coastal bryconine clade (without *B*. *opalinus*) on the concatenated tree of Abe and collegues (Fig 3 from Abe *et al*. [14]). Low levels of phylogenetic support among eastern coastal bryconins are more evident on Abe and colleagues analysis (Fig 4 from Abe *et al*. [14]) and *B*. *nattereri* and *B*. *opalinus* sister species status may be the result of long-branch attraction between them. Likewise, our analysis showed a more resolved phylogenetic relationship among all eastern coastal basin bryconine species, including *B*. *insignis*, *B*. *ferox*, *B*. *vermelha* and *H*. *wheatlandii*, which is unresolved in Abe *et al*. [14].

The presence of one well-defined clade composed exclusively by species present in the eastern coastal river basins, seems to be the result of the long independent paleohydrological history of these watersheds. The coastal basins of eastern Brazil are isolated from the continental watersheds by Serra da Mantiqueira and Serra do Espinhaço, which represent the boundaries of the distribution ranges of various fish species [8, 9]. It has been reported that the patterns of cytogenetic and molecular variation in the predator *Hoplias malabaricus* are consistent with the paleohydrological history of the coastal basins of eastern Brazil [10], although this species also shows evidence of geodispersal between coastal and other continental basins [16]. The pattern of *Brycon* distribution in the coastal basins partially fits the subdivision of the areas of endemism among the Paraíba do Sul and Doce Rivers proposed by Carvalho [65]. Furthermore, Menezes [66] proposed that the Atlantic coastal drainages can be divided into three regions of endemism: the Northern region of the Doce River to the mouth of the Jequitinhonha River; a Central region from the Cubatão River to the Itabapoana River to the north, characterized by the occurrence of *Oligosarcus hepsetus*; and a Southern region, characterized by the presence of *Oligosarcus jenynsii* and *Oligosarcus robustus*. In addition, Abell *et al*. [67] also classified drainages into six ecoregions based on the compositions of species in different taxonomic levels: Northeast Atlantic, Paraíba do Sul, Fluminense, Ribeira de Iguape, Southeastern Atlantic, Tramandaí-Mampituba, and Laguna dos Patos. However, Buckup [68] indicated that the limits and relations of endemism along the Brazilian Shield are not yet well defined.

The phylogeographic analyses carried out in the present study based on the cytogenetic and molecular data also shed light on the taxonomic status of some Bryconidae. According to the phylogenetic hypothesis, *H*. *wheatlandii* appears to be closely related to *B*. *ferox* from the Mucuri River, which corroborates the evidence that *Brycon* is a paraphyletic group [11, 12, 14]. The high degree of morphological divergence seen in *H*. *wheatlandii* relative to its sister species has been considered as an example of adaptive radiation [69], which in this case seems to be unrelated to the extinction of intermediate forms. The position of *B*. *devillei* and *B*. *insignis* as reciprocally monophyletic and allopatric species is reinforced by the high degree of cytogenetic divergence between them.

Specimens of *B*. *devillei* from the Doce and the Mucuri rivers shared the same cytogenetic and molecular characters, indicating that these populations belong to the same species. This is especially informative for the taxonomy of this species. Although Lima *et al*. [1] state that the precise type locality of *B*. *devillei* is unknown, the Mucuri River drains into the Atlantic Ocean in the southern tip of the Bahia State, which is consistent with the original Castelnau’s description [1]. The close relatedness of the Doce and Mucuri populations can be explained by the recently paleohydrology of these watersheds. As a result of eustatic sea level variations during the periods of glacial maximum, the existing drainage of the Doce and Mucuri rivers probably converged in paleo channels that are currently represented by isobaths of 60 m and surrounded by 30-m isobaths [70]. These Pleistocene paleochannels are delimited to the south by the mouth of the Doce River and to the north by Abrolhos Formation, and explain the patterns of distribution and phylogeography of the freshwater fish fauna [10] unrelated to Bryconidae.

The results of the present study demonstrate that the Bryconinae from the coastal basins of southeastern Brazil form a phylogenetic and phylogeographic unit. The cytogenetic characteristics that differentiate these fish from their continental congeners are: presence of a pair of subtelocentric chromosomes bearing NORs (vs. submetacentric) and the absence of equilocal heterochromatic blocks in their first chromosome pair; this monophyly is strongly supported by molecular data. Our results partially corroborate the “*Brycon acuminatus*” group proposed by Howes in 1982 [2], keeping *B*. *devillei*, *B*. *ferox*, and *B*. *insignis;* and including *B*. *opalinus* (which was a member of Owen’s “falcatus” group), *B*. *vermelha* (described by Lima and Castro [4]), and *H*. *weatlandii* (formerly considered unrelated to *Brycon* [11]) whereas it excludes *B*. *nattereri*.

## Supporting Information

S1 Fig Chromosome spread from Ag-NOR banding protocols presented in this work. *Brycon devillei* (a); *Brycon ferox* (b); *Brycon insignis* (c); *Brycon opalinus* (d), and *Brycon vermelha* (e).
(TIF)
