# Supplementary figures and images for: Cytogenetic and Molecular Data Demonstrate that the Bryconinae (Ostariophysi, Bryconidae) Species from Southeastern Brazil Form a Phylogenetic and Phylogeographic Unit
Source: PLoS One. 2015 Sep 15;10(9):e0137843. doi: 10.1371/journal.pone.0137843 (PMC4570709; doi:10.1371/journal.pone.0137843)

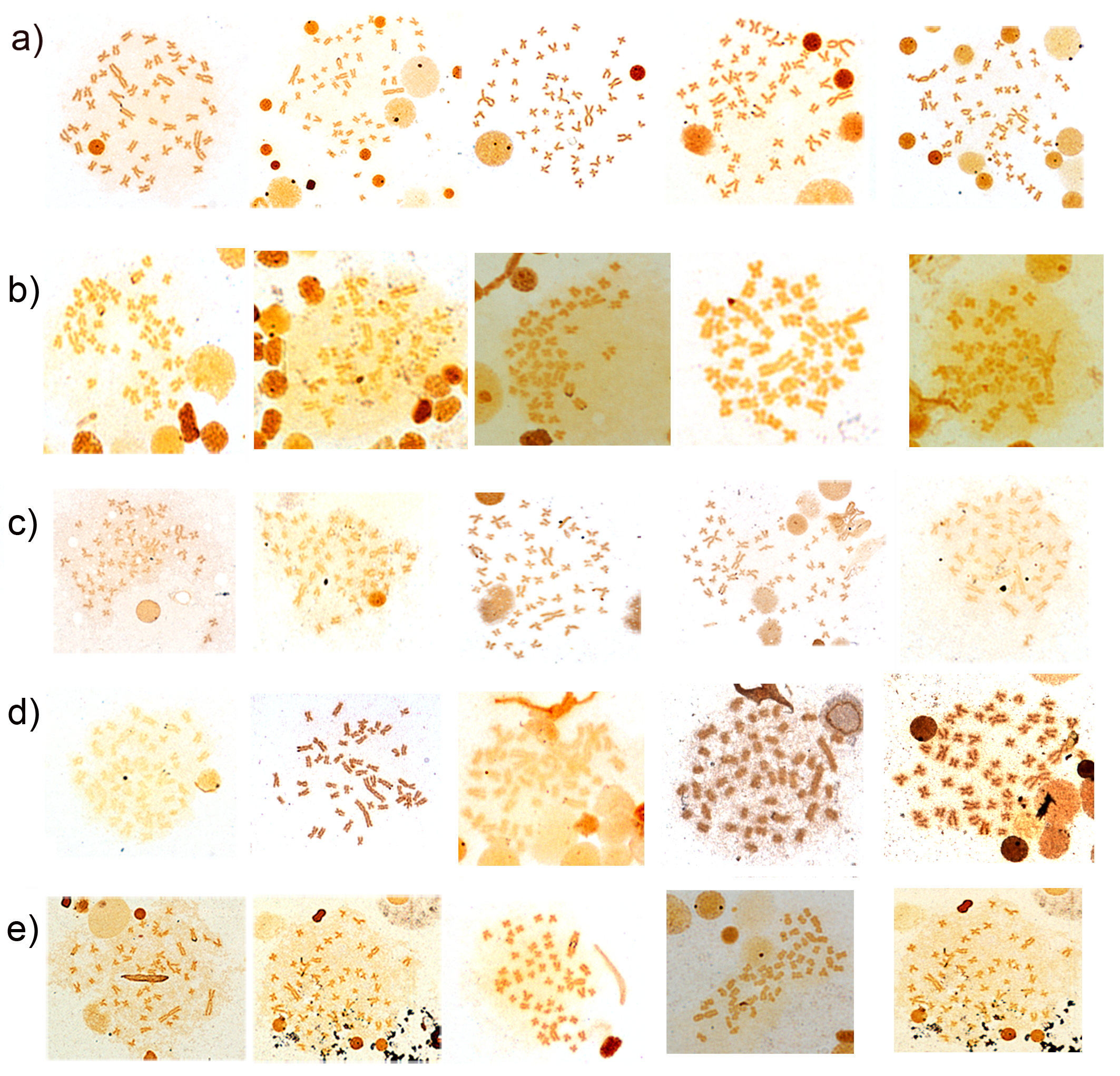

Supplement: S1 Fig — Brycon devillei (a); Brycon ferox (b); Brycon insignis (c); Brycon opalinus (d), and Brycon vermelha (e). (TIF) [file pone.0137843.s001.tif]
